# Effects of plant productivity and species richness on the drought response of soil respiration in temperate grasslands

**DOI:** 10.1371/journal.pone.0209031

**Published:** 2018-12-21

**Authors:** Susanne Burri, Pascal A. Niklaus, Karin Grassow, Nina Buchmann, Ansgar Kahmen

**Affiliations:** 1 Department of Environmental Sciences – Botany, University of Basel, Basel, Switzerland; 2 Institute of Agricultural Sciences, ETH Zurich, Zurich, Switzerland; 3 Department of Evolutionary Biology and Environmental Studies, University of Zurich, Zurich, Switzerland; 4 Fritz-Haber-Institut der Max-Planck-Gesellschaft, Berlin, Germany; Chinese Academy of Forestry, CHINA

## Abstract

Soil respiration plays a crucial role in global carbon cycling. While the response of soil respiration to abiotic drivers like soil temperature and moisture is fairly well understood, less is known about the effects of biotic drivers, such as plant above- and belowground productivity or plant diversity, and their interactions with abiotic drivers on soil respiration. Thus, current predictions of soil respiration to summer droughts might miss relevant biological drivers and their interactions with abiotic drivers. Since drought events are expected to increase in Central Europe in the future, we simulated early summer drought using rainout shelters at 19 grassland sites, which differed in plant productivity and species richness in central Germany in 2002 and 2003. We tested the potentially interacting effects of drought with biotic drivers, i.e. annual above-ground productivity, species richness and root biomass, on the drought response of soil respiration in temperate grasslands. In both years, drought led to a significant reduction in soil respiration. The drought-induced reduction in soil respiration was largely driven by the reduction in above-ground productivity in response to drought. The extent of the drought response of soil respiration was dependent on the species richness level of the site and this interacting effect was explainable by the variation in root biomass (root biomass and species richness were positively correlated). Our findings highlight the importance of biotic drivers for the quantification of the drought response of soil respiration in grasslands.

## Introduction

Soil-respired carbon dioxide (CO_2_) integrates the release of CO_2_ from soils via root (autotrophic) or microbial (heterotrophic) respiration and strongly depends on environmental conditions, with soil temperature and moisture being the main abiotic drivers [1]. While the response of soil respiration to soil temperature is often described as an exponential relationship [2], the influence of soil moisture on soil respiration is usually described by a plateau-shaped curve [3, 4]. Interestingly, known soil temperature and soil moisture response functions often fail to predict the impact of climate change on soil respiration [4, 5]. Thus, we are currently unable to reliably predict effects of drought on soil respiration, which is critical for understanding climate change effects on the carbon (C) budget of terrestrial ecosystems.

A possible reason why soil temperature and soil moisture functions alone are insufficient for predicting the response of soil respiration to drought is the fact that soil respiration is additionally driven by above-ground processes such as photosynthesis and above-ground productivity [6–10]. Moreover, drought stress can reduce the coupling of photosynthesis and soil respiration by reducing the carbohydrate supply to below-ground compartments [11–15]. The tight coupling between above- and below-ground processes is currently insufficiently integrated into the response functions of soil respiration to drought. This could explain why model predictions of climate change effects on soil respiration are still relatively weak [5]. In particular in grasslands, with their large contribution of below-ground productivity to their overall carbon budget, the coupling to above-ground processes cannot be neglected when the response of soil respiration to environmental variability is assessed.

A further factor driving the functioning of grasslands is biodiversity, often quantified as species richness (see e.g. Balvanera et al. [16], Cardinale et al. [17]). Although there is an increasing evidence that above-ground productivity as well as the resilience and resistance of productivity to disturbances increases with species diversity (e.g. Lehmann and Tilman [18], Kahmen et al. [19], Proulx et al. [20], Isbell et al. [21]), it is unclear if and how productivity and/or plant diversity affect the response of soil respiration to drought [22]. For example, Craine et al. [23] found a positive effect of plant diversity on soil respiration in grasslands, while in the study of Dias et al. [24], this effect has mainly been attributed to changes in productivity as species richness and productivity were positively correlated. Yet, another study has reported no effect of plant diversity on soil respiration [25], while Johnson et al. [26] have found that plant community composition but not biodiversity or above-ground biomass were driving soil respiration in mesocosm communities. In summary, these studies indicate that productivity and biodiversity can be important drivers of soil respiration in grasslands, but large scale experiments, which allow studying the effect of biotic drivers on soil respiration in response to drought, are still missing.

Here, we investigated the effect of drought on soil respiration in 19 grasslands sites in central Germany during two years (2002 and 2003). The grasslands differed in their annual productivity and species richness, but were comparable in their climatic conditions. Early summer drought was simulated by using rainout shelters. Thus, our objectives were i) to assess soil respiration in 19 different grasslands varying in above-ground productivity and species richness over two years, ii) to determine the effect of drought on soil respiration in these 19 different grasslands, and iii) to investigate the potentially interacting effects of biotic drivers (annual above-ground productivity, species richness and root biomass) on the drought response of soil respiration. Our setup was unique as it allowed for the first time to experimentally assess drivers other than soil temperature and soil moisture on soil respiration during drought across a large number of different grassland sites.

## Materials and methods

### Study sites and experimental design

The drought experiments for this study were conducted in 19 grassland sites located in the Thüringer Schiefergebirge/Frankenwald in central Germany at the Thuringian/Bavarian border. Each site was on private land and the owner of the respective grassland site gave permission to conduct the study on this site. The sites at the plateau-like mountain range were located between 550 and 720 m a.s.l, within an area of roughly 400 km^2^. The distance between the two sites furthest apart was 27 km (Table 1). The climatic conditions across the 19 sites were comparable, but the grasslands differed in annual productivity and plant species richness. All grasslands were extensively managed, unfertilized and cut twice a year (but not grazed). The soils were carbonate-free and nutrient-poor brown earths / cambisols, with pH ranging from 4.4 to 6.1 and soil C concentrations between 30 and 75 mg C g^-1^, for further details see Kahmen et al. [27]. Groundwater was too far away to influence our sites.

**Table 1:** Location of the 19 grassland sites.

Site number	Latitude	Longitude	Altitude (m a.sl)
1	50.4111885	11.6271771	639
2	50.4093085	11.6268261	654
3	50.4097615	11.6279339	641
4	50.4568025	11.5921776	631
5	50.4620167	11.5973717	604
6	50.4824550	11.5783183	642
7	50.4790458	11.5560044	558
8	50.4672671	11.4958875	681
9	50.4252458	11.5078731	721
10	50.4033153	11.4450283	683
11	50.3848272	11.4459190	619
12	50.4085589	11.3796513	630
13	50.4158451	11.3855640	643
14	50.4525175	11.4050836	687
15	50.4487572	11.4081156	692
16	50.4412033	11.3633738	651
17	50.4391912	11.3378938	654
18	50.4581812	11.3480215	686
19	50.4781784	11.2627800	668

The geographical position of the 19 grassland sites. The sites were located between about 550 and 720 m a.s.l, within an area of roughly 400 km2.

Two 5 x 5 m plots were established at each site. One of the plots served as a control plot and the other as a drought plot. For each plot, a core area of 1 x 2m was established in the center of the plot, where soil respiration measurements and vegetation sampling were performed.

On the drought plots, tunnel-shaped rainout shelters were installed that were 2.3 m in height and covered an area of 3 x 3.5 m. The steel frames of the shelters were covered with transparent plastic foil, excluding precipitation for 6–7 weeks from spring to early summer in 2002 and 2003 (2002: 16–21 May to 18–20 June; 2003: 23–27 April to 10–13 June). The plastic foil allowed 90% of the photosynthetically active radiation to pass through (Cello Flex 4TT, Prosyn Polyane, St Chamond, France). To guarantee sufficient air circulation and to avoid heating, the shelters were open at the two ends of the tunnel [19].

At six of the 19 sites, meteorological variables, namely air temperature (at 60 cm height) as well as soil temperature and soil moisture (at 5–10 cm soil depth), were measured continuously and logged hourly (Campbell Scientific Inc., Logan, UT, USA) on both control and drought plots from May 2002 to December 2003 (covering the most active period of these grassland sites). We refer to the difference in soil moisture between drought and control plots as ΔSM (SM drought—control) from hereon, with negative ΔSM representing reduced soil moisture due to the drought treatment. Precipitation data from a nearby flux tower Wetzstein (DE-Wet) had been collected within the project CarboEurope (EU-FP6) and were downloaded from the European Fluxes Database Cluster.

### Measurement and modeling of soil respiration rates

Three soil collars (diameter 10.3 cm) were installed on each control and drought plot at each site (reaching 1–2 cm into the soil) to measure soil respiration at the same locations over the two years of the experiment. The above-ground part of the vegetation inside the collars was clipped regularly in order to exclude above-ground respiration from the measurement. Since the root system in grassland spreads out over larger areas than the small area covered by the soil collars used, our soil respiration measurement also integrates the root respiration from plants growing around the collars, especially since the insertion depth of the collars was very low.

Soil respiration was measured every 2–3 weeks during the most active periods of these grasslands (2002: June to November; 2003: April to October), using the chamber system LI-6400-09 connected to the LI-6400 IRGA (Licor Inc., Lincoln, NE, USA). The sequence of all sites within each of these measurement cycles, which typically took 2–3 days, was changed each time, in order to avoid always measuring the same site at the same time of the day. This approach also took diel variations of soil respiration into account since each soil respiration measurement was complemented by manual measurements of soil temperature and soil moisture (ThetaProbe, Delta-T Devices, Cambridge). The three measurements per plot were averaged, quality-checked and filtered for further analyses. Hence, we excluded measurements that were 1.5 times the interquartile range below the first quartile or above the third quartile of all soil respiration data, or if their standard deviations were 1.5 times the interquartile range below the first quartile or above the third quartile of all standard deviations of measured soil respiration rates. We still retained 95% of the original data after filtering.

In order to test the effect biotic drivers on soil respiration as well as on its response to drought, we needed one value of soil respiration per plot and year that we could relate to the discontinuous biotic drivers assessed in this study. In order to reach this aim, we generated a continuous data set of hourly soil respiration rates per plot (i.e. SR_hourly_) for the two years of our study, which we could then sum up to one value (see details below). This data set was created by soil respiration modelling based on our own soil climate (i.e. temperature and soil moisture) as well as our manual soil respiration measurements. The soil respiration modelling included as a first step the data assimilation of the continuously (six sites) as well as the manually (19 sites) measured soil climate variables, followed by the modeling of soil respiration rates on an hourly time scale.

The two different data sets for soil temperature and soil moisture (manual measurements at 19 sites, continuous measurements at six sites, measurements from May 2002 until December 2003) were assimilated into a continuous soil climate dataset for all 19 sites. This first required the linear interpolation of hourly soil temperature and soil moisture data at the 13 sites where soil climate data had not been logged hourly, as the distance-weighted mean of the measurements of the six logger sites (weights were defined as the inverse distance to the six logger sites). In a second step, we calculated linear regressions per site (including both control and drought plots) between the manually measured data and the continuous data (either measured at six sites or generated at 13 sites) at the time of the manual measurement resulting in site-wise coefficients. Using these site-specific coefficients, the continuous data of each plot (separately for control and drought plots) was linearly adjusted to site-specific conditions. This resulted in an assimilated continuous soil climate data set for each of the 19 grassland sites from May 2002 until December 2003.

In the following step, soil respiration rates were modeled on an hourly time scale by combining an exponential temperature function [28] with a soil moisture dependency [29]:

$$SR_{hourly}=\frac{SR_{ref}\cdot e^{E_{0}\left(\frac{1}{56.02}-\frac{1}{ST+46.02}\right)}}{1+e^{\left(a+b*SM\right)}}$$ (1)

Where *SR* refers to soil respiration (μmol m^-2^ s^-1^), *SR*_*ref*_ (μmol m^-2^ s^-1^) is soil respiration at 10°C if soil moisture is not limiting, *E*_*0*_ is the parameter for temperature dependency (K), *ST* is soil temperature (K), and *SM* is soil moisture (Vol.%), *a* and *b* are fitted parameters.

The soil respiration model was fitted plot-wise (i.e. separately for each control and drought plot) for each site including both years, because we assumed similar soil respiration dependencies on abiotic factors for both years. The fitting was done by a constrained non-linear regression, yielding *SR*_*ref*_, *a* and *b* for each plot and site (S1 Table). Finally, continuous hourly soil respiration rates were successfully predicted using the continuous soil climate data set from May 2002 until December 2003 separately for each plot at each site.

For the final analysis, we needed one measure of soil respiration per plot, which was comparable between the two years (annual soil respiration rates were not possible, because measurements only started in May 2002). Thus in a final step, soil respiration sums from the beginning of the respective drought treatment until the end of each year were calculated for each year separately, which were then normalized over the number of days these periods included. This in turn resulted in mean daily soil respiration rates (SR_daily_). In order to compare our modeling approach to previous studies, annuals sums of soil respiration for 2003 were calculated (only in 2003 a full year of data was available). ΔSR always refers to the difference of soil respiration rates between drought and control plots (SR drought and control), thus negative ΔSR values depict reduced soil respiration rates due to the drought treatment.

### Sampling of annual above-ground productivity, species richness and standing root biomass

Above-ground biomass was harvested twice per year, with the first harvest shortly after shelter removal in June and the second one in early September. Both harvests were summed up to estimate annual above-ground productivity. Each above-ground biomass measurement was an average of four 25 x 50 cm subsamples per plot. Shoots were clipped 2 cm above ground, dried (48 h at 60°C) and weighed. In mid-June 2002, before the first above-ground biomass harvest, the species number, i.e. species richness, was assessed on each control and drought plot. Standing below-ground biomass in the top 10 cm (root biomass) was measured once in June 2002 shortly after shelter removal (at the same four subplots used for the above-ground biomass harvest) using a soil core auger of (4.3 cm diameter). Ravenek et al. (2014) report from an biodiversity experiment (Jena Experiment, [30]) that root mass density in 78 experimental grasslands plots over 8 years decreased significantly with depth (0 to 40 cm depth), showing a mean rooting depth over all plots of 9.7 cm ± 0.2 (SE). They did not find any differences of vertical root distribution with increasing species richness. Thus, we have strong confidence that we cover about 50% of the main rooting system when sampling 0 to 10 cm. Roots were washed on a 0.5 mm mesh, dried (48 h at 60°C) and weighed.

### Statistical analyses

All data were analyzed by analysis of variance using hierarchical linear models fitted with the aov function of R (R 3.2, http://r-project.org). All respiration- and biomass data were log-transformed prior to analysis to obtain normal, homoscedastic residuals and to test for differences in relative effects of the treatment (e.g. drought × year or drought × covariate interactions). As a first step, we tested for the effects of the drought treatment on soil respiration, soil moisture and biotic drivers for the two years separately. The model consisted of the fixed terms site, followed by treatment.

For soil respiration, we then analyzed the data of the two years together. For this analysis, we included the fixed terms site, treatment, year and the interaction drought × year, while plot was fitted as error term (Error option of aov). While the drought treatment was tested using the among-plot variance as error term, the drought × year interaction was tested using the residual variance (which is equivalent to plot × year) as error term.

To explore potential mechanisms linked to biotic drivers underlying drought effects, we then added covariates to these models (annual above-ground productivity, standing root biomass, or species richness). With the inclusion of covariates, the model terms, were no longer orthogonal and we therefore fitted all models by REML (Restricted Maximum Likelyhood). To separate across and within site (i.e. largely treatment-related) effects of these covariates, we first fitted their site average followed by their plot-level value. This allowed to first test for effects of covariates among sites (i.e. they explained differences in average site responses, irrespective of the drought treatment), and then, in addition, effects of the same covariate on variation among plots. In these models, the order of factors matters, and this is “by design” because we aimed at testing for effects of drought after adjusting the data for effects of the covariate. The reason is that covariates may have different effects among and within sites, for various reasons. For example, a covariate (e.g. species richness) may explain differences in soil respiration among sites, irrespective of the experimental treatment “drought”. On the other hand, plots may differ in the covariate (e.g. diversity) within site, and this effect may be driven by other mechanisms. The analysis we have chosen allows to separate these within and across site effects, which is important. It allows to test for residual drought effects, i.e. on data adjusted for the covariate in a clean way, not mixing up the among site effect and the within site effect (i.e. the drought effect).

## Results

### Meteorological conditions on control plots

**Fig 1:**
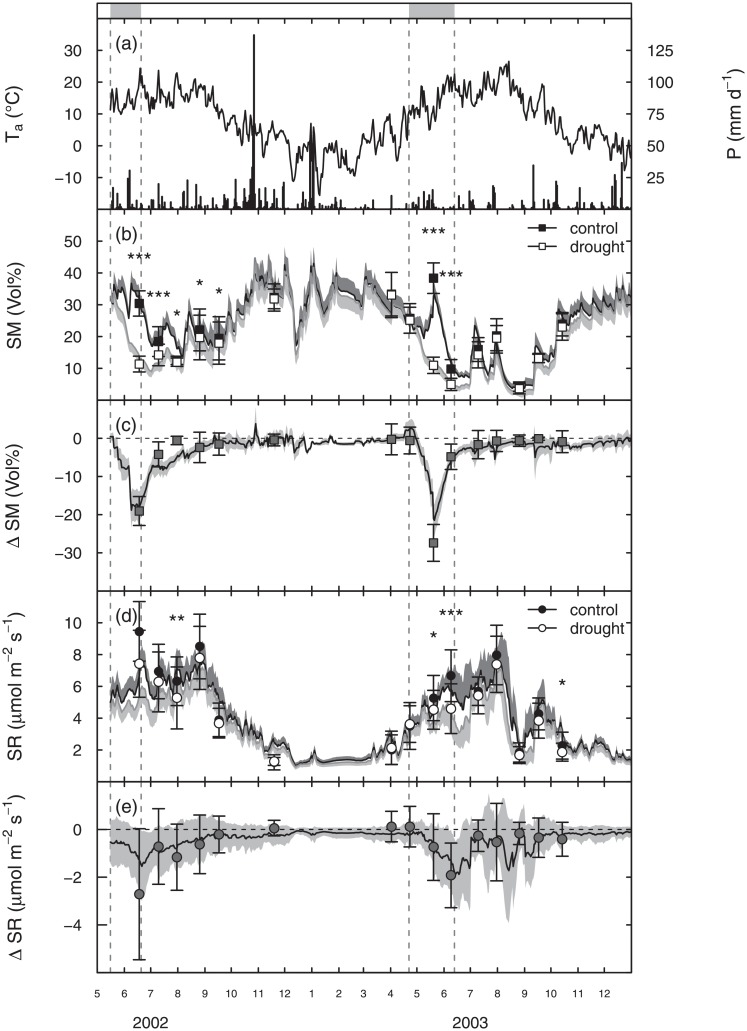
Measurements from May 2002 to December 2003. (a) daily mean air temperature (Ta, n = 6, from sites where data loggers were installed) and precipitation (P, daily sums of precipitation at the meteorological station Wetzstein); (b) mean manually measured soil moisture (SM), daily mean values of assimilated continuous SM (mean ± SD, n = 19) and (c) mean difference in SM between control and drought plots (ΔSM); (d) mean manually measured soil respiration (SR), daily mean values of the modelled seasonal courses of SR (mean ± SD, n = 19) and e) the difference in SR between control and drought plots (ΔSR). Grey rectangles in the top panel mark the shelter periods (continued by dashed lines into the lower panels). For clarity, SD is shown in one direction only. Numbers on time axis are months of the year. Stars show levels of significance: *p < 0.05, **p < 0.01, ***p < 0.001 (tested by ANOVA).

Air temperatures during the 2-year experiment were very similar in 2002 and 2003, with mean air temperatures during the time of shelter installation on control plots of 14.4°C (2002) and 13.8°C (2003), respectively (Fig 1a). Soil moisture in control plots followed similar seasonal courses across all sites and the standard deviation of the assimilated continuous soil moisture data across the 19 sites (aggregated to daily mean values) did not exceed 6 vol% within the measurement period of two years (Fig 1b). The natural heat wave in August 2003, which hit large parts of Europe as an extreme event, also affected our sites: Average daily mean temperatures were 2.6°C higher in August 2003 than in August 2002 (2002: 17.3°C, 2003: 19.9°C) and maximum recorded air temperatures were 34.9°C in 2003 in comparison to 27.6°C in 2002. In addition, the natural drought and heat wave in August 2003 drastically reduced soil moisture on all plots (Fig 1b). However, this extreme event occurred several weeks after shelter removal and did not overlap with our drought experiment.

### Soil respiration and biotic drivers on control plots

**Fig 2:**
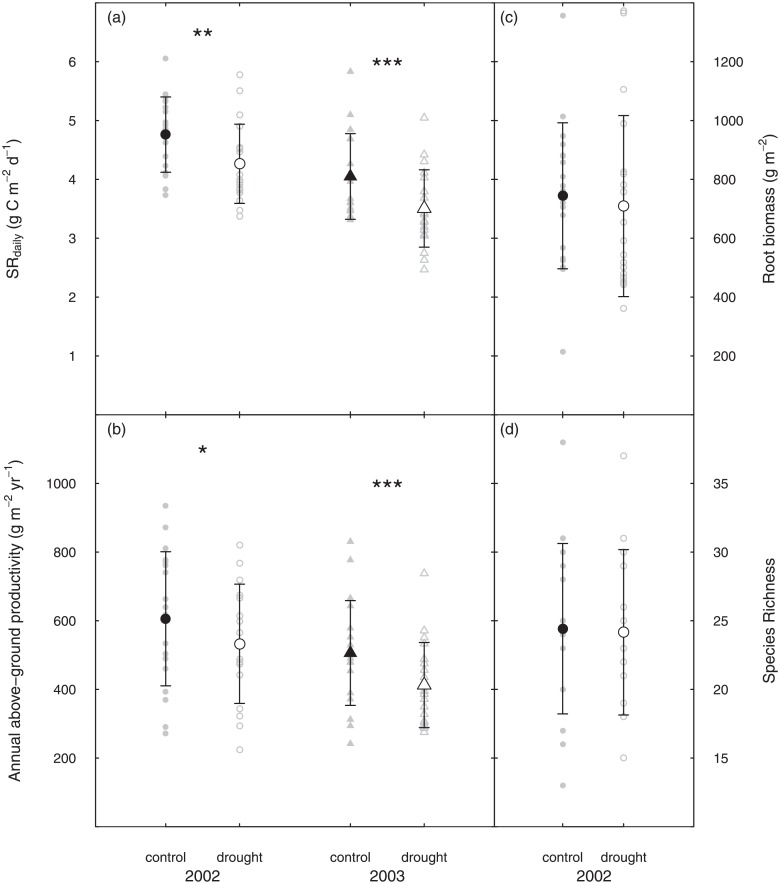
Soil respiration and biotic drivers under control and drought conditions. (a) mean daily soil respiration (SRdaily) and (b) mean annual above-ground productivity (c) standing root biomass and (d) species richness in 2002 (circles) and 2003 (triangles) for control (filled symbols) and drought conditions (open symbols) (mean ± SD, n = 19). Underlying grey symbols show single values. Stars indicate levels of significance: *p < 0.05, **p < 0.01, ***p < 0.001 (tested by ANOVA).

Both manually recorded and modeled soil respiration rates varied considerably among the 19 grassland sites (Fig 1d). Cumulative annual sums of soil respiration in 2003 ranged from 1023 to 1720 g C m^-2^ yr^-1^ under control conditions, with a mean annual C release by soil respiration of 1224 ± 195 g C m^-2^ yr^-1^ (mean ± SD). Calculated mean daily soil respiration rates (SR_daily_) on control plots across the 19 sites ranged from 3.3 to 6.1 g C m^-2^ d^-1^ over the two years 2002 and 2003. Mean SR_daily_ across the 19 grassland sites were 17% (p<0.001) lower on control plots in 2003 compared to 2002 (2002: 4.8±0.6 g C m^-2^ d^-1^, 2003: 4.0±0.7 g C m^-2^ d^-1^, mean ± SD, Fig 2a).

Annual above-ground productivity on control plots across the 19 sites ranged from 242 to 935 g m^-2^yr^-1^ during the two years 2002 and 2003. Similarly to SR_daily_, mean annual above-ground productivity was 17% (p<0.01) lower on control plots in 2003 compared to 2002 (2002: 606±195 g m^-2^yr^-1^, 2003: 506±153 g m^-2^ yr^-1^ in 2003, mean ± SD, Fig 2b). Species richness and standing root biomass were only assessed in 2002. Species richness ranged from 13 to 38 species on control plots, mean standing root biomasson control plots was 744±248 g m^-2^ (mean ± SD) in 2002.

**Fig 3:**
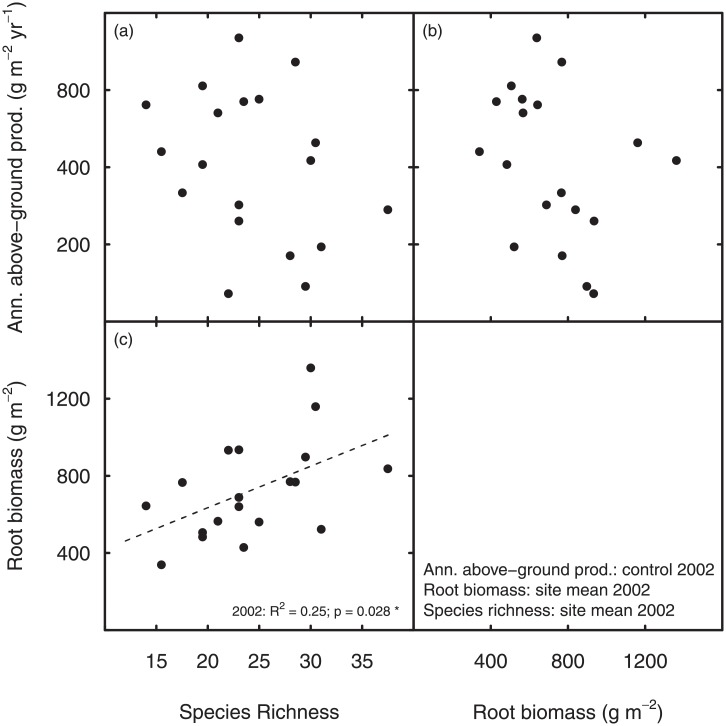
Correlations between biotic drivers. Scatterplot matrix for correlations between annual above-ground productivity (under control conditions), species richness and standing root biomass in 2002. Since neither species richness nor standing root biomass showed significant changes in response to the drought treatment, site means from control and drought plots are shown.

Annual above-ground productivity under control conditions was unrelated to species richness (Fig 3a) and root biomass (Fig 3b) at the 19 grassland sites in 2002. However, species richness and standing root biomass were significantly positively correlated with each other (r = 0.5, P = 0.02), when both control and drought plots were pooled (Fig 3c), which was possible because drought affected none of them.

### Drought effects

#### Drought simulation by rainout shelters

The rainout shelters successfully simulated early summer drought during the treatment phase. Soil moisture was strongly reduced by the rainout shelters both in 2002 and 2003 (Fig 1b). At the peak of the drought treatment, drought and control plots differed by 19.1 ± 3.8 vol% in 2002 and by 27.4 ± 4.8 vol% in 2003 (mean ± SD, Fig 1c). In 2002, the difference in soil moisture was still significant three months after shelter removal (Fig 1b). On the other hand, in 2003, these differences in soil moisture disappeared shortly after shelter removal, probably due to much lower soil moisture levels at control plots during the treatment phase in 2003 compared to 2002 (Fig 1b and 1c).

Addressing potential shelter artefacts on micrometeorology, we generally observed a mean decrease in daily maximum air temperatures (mean over the shelter period, 2002: -0.2°C, 2003: -0.4°C) and a mean increase in daily minimum air temperatures (mean over the shelter period, 2002: +0.3°C; 2003: +0.3°C) caused by the rainout shelters. However, overall mean air temperatures over the shelter period were only slightly changed by +0.08°C in 2002 and +0.03°C in 2003 on drought plots compared to control plots. Considering the temperature sensitivity of soil respiration and our model results (Eq 1, S1 Table) at a given soil moisture of 30 vol%, we found that a change of 1°C would translate to a maximum increase of 0.5 μmol m^-2^ s^-1^ at a soil temperature of 10°C and to 0.7 μmol m^-2^ s^-1^ at a soil temperature of 20°C. Thus, potential experimental biases seem small and well within the natural spatial variations among sites indicating that the observed effect on soil respiration in our experiment was mainly caused by drought.

#### Impact of drought on soil respiration

Drought reduced soil respiration during the shelter phase in both years, but the reduction across all grassland sites was significant in 2003 only (Fig 1d). The strongest reduction in soil respiration occurred always at the end of the drought period (2002: -1.54 ± 1.46 μmol m^-2^ s^-1^ (average reduction of 20% compared to control plots), 2003: -1.89 ± 1.40 μmol m^-2 s-1^ (average reduction of 34% compared to control plots), mean ± SD, Fig 1e). Similar to soil moisture recovery, recovery of soil respiration was slower in 2002 than in 2003. Overall, the standard deviations of ΔSR varied considerably among the different grassland sites, indicating different responses of soil respiration to drought across sites (Fig 1e).

SR_daily_ on drought plots ranged from 2.5 to 5.8 g C m^-2^ d^-1^ during these two years. Mean SR_daily_ across the 19 grassland sites was 22% higher (P<0.001) on drought plots in 2002 compared to 2003 (2002: 4.3±0.7 g C m^-2^ d^-1^, 2003: 3.5±0.7 g C m^-2^ d^-1^, mean ± SD, Fig 2a). The drought treatment reduced SR_daily_ by 10% in 2002 (P<0.01) and 13% in 2003 (P<0.001) (Fig 2a).

#### Impact of drought on biotic drivers

Mean annual above-ground productivity on drought plots across the 19 grasslands ranged from 224 to 820 g m^-2^yr^-1^ over the two years 2002 and 2003. It was 29% higher (P<0.001) on drought plots in 2002 compared to 2003 (2002: 533±174 g m^-2^yr^-1^, 2003: 413±124 g m^-2^yr^-1^ in 2003, mean ± SD, Fig 2b). Drought decreased annual above-ground productivity significantly by 12% (p<0.05)in 2002 and 18% (p<0.001) in 2003 (Fig 2b).

Mean standing root biomass on drought plots was 709±308 g m^-2^ (mean ± SD) and species richness ranged from 15 to 37 species in 2002. Neither standing root biomass nor species richness were significantly affected by the drought treatment. (Fig 2c and 2d).

#### Interaction of drought and biotic drivers on soil respiration

Including annual above-ground productivity as a covariate into our models (Table 2) showed annual above-ground productivity averaged per site explained only a small fraction of the variation in SR_daily_ across sites and across both years (P = 0.09 in REML model). However, highly significant effects of plot-level annual above-ground productivity on SR_daily_ were found within site (P<0.001) (i.e. treatment-related). Drought effects were highly significant (P<0.001) when fitted before plot-level annual above-ground productivity (Table 2A), but they were only marginally significant (P = 0.08) when fitted after plot-level annual above-ground productivity (Table 2B). This suggests that drought effects on soil respiration were largely induced by reductions in above-ground productivity in response to drought. Conversely, effects of plot-level above-ground productivity on soil respiration remained significant (P<0.001) when fitted after drought, indicating also significant but drought-unrelated effects of above-ground productivity on soil respiration. No dependencies of these effects on year were found.

**Table 2:** Annual above-ground productivity as a covariate.

	Df	denDF	F.inc	*P* value	
**A Drought treatment fitted before above-ground productivity**					
(Intercept)	1	17.0	2310	<0.001	***
Above-ground prod. (Site average)	1	17.0	3	0.090	
Treatment	1	16.9	22	<0.001	***
Above-ground prod. (Plot level)	1	44.2	44	<0.001	***
Year	1	42.2	36	<0.001	***
Above-ground prod. (Plot level) x Treatment	1	31.7	1	0.393	
Treatment x Year	1	44.3	1	0.277	
**B Drought treatment fitted after above-ground productivity**					
(Intercept)	1	17.0	2310	<0.001	***
Above-ground prod. (Site average)	1	17.0	3	0.090	
Above-ground prod. (Plot level)	1	50.6	62	<0.001	***
Treatment	1	21.6	4	0.073	
Year	1	42.2	36	<0.001	***
Above-ground prod. (Plot level) x Treatment	1	31.7	1	0.393	
Treatment x Year	1	44.3	1	0.277	

Summarized results (degrees of freedom (Df), denominator degrees of freedom (denDF), F statistic for the incremental sum of squares (F.inc) significance levels (P value) from testing the effects of drought (Treatment), year and the covariate above-ground productivity on soil respiration (SRdaily). The difference in results between the two models (A and B) is marked by grey shading. Site and plot were specified as random effects. Stars show significance level:

*** p < 0.001.

**Fig 4:**
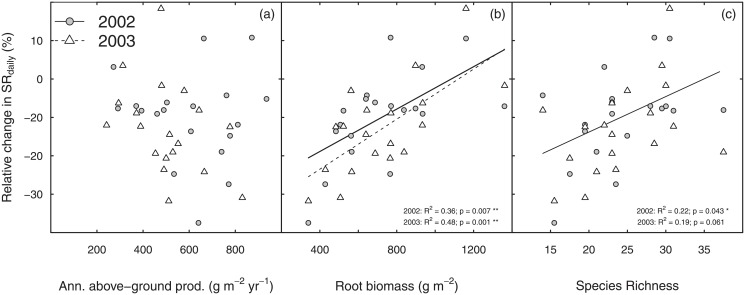
Relative change in soil respiration in relation to biotic drivers. Relative reduction of mean daily sums of soil respiration (SR_daily_) in 2002 (circles) and 2003 (triangles) in response to the drought treatment correlated with (a) annual above-ground productivity under control conditions (b) root biomass and (c) species richness. For species richness and root biomass site means from 2002 are shown. Stars indicate levels of significance: *p < 0.05, **p < 0.01 (tested by ANOVA). Note that the interactions terms in our REML approach tested for the same relative effects because the data was log-transformed prior to analysis.

Separate analyses of data for both years showed similar results. Annual above-ground productivity averaged per site explained some variation in SR_daily_ in 2002 (P<0.02) but not in 2003 (P = 0.3). Drought-effects on SR_daily_ were significant in 2002 (P<0.01) and 2003 (P<0.001) when fitted before plot-level annual above-ground productivity; their significance dropped substantially when fitted after plot-level annual above-ground productivity (2002: P = 0.01; 2003: P = 0.03), indicating a strong effect of drought-related above-ground biomass reductions on soil respiration. We found no significant interaction effect of drought and above-ground productivity and hence also the relative reduction in soil respiration in response to drought (i.e. the percentual decrease in SRdaily from control to drought plots as a measure for the susceptibility of the respective grassland to drought) was independent of above-ground productivity. This suggests that annual above-ground productivity at the grasslands had no effect on the susceptibility of grasslands to drought (Fig 4a).

The same analysis for standing root biomass did not explain any significant fraction of variation in SR_daily_ across sites nor within site, irrespective of whether this covariate was fitted before or after drought. However, we found a significant interaction of drought and standing root biomass (P<0.001), which is also shown by the significant positive relationship between ΔSR_daily_ and standing root biomass (p<0.01 both in 2002 and 2003, Fig 4b). Hence, the impact of drought on soil respiration decreased with increasing root biomass.

Including species richness as a covariate did not explain any significant variation in SR_daily_ neither across sites nor within site. However, a significant interaction between species richness and drought was found (P<0.05) similar to the interaction of standing root biomass and drought. Similar to standing root biomass, the relationship between ΔSR_daily_ and species richness was positive (Fig 4c, p<0.05 in 2002, only a tendency in 2003). This suggests that grasslands with higher species richness exhibited smaller changes in soil respiration than grasslands with lower species richness. This interaction between species richness and drought could fully be explained by variations in standing root biomass (Species richness × drought n.s. (P = 0.6) when fitted after standing root biomass × drought).

## Discussion

The 19 grassland sites were comparable in their climatological conditions and the variation in soil moisture across the 19 sites was low enough in order to fulfill an important prerequisite of our study: The 19 sites needed to be comparable in their abiotic conditions in order to analyze the effect of biotic drivers on the drought response of soil respiration. The simulation of summer drought, another prerequisite of our study, was successful and potential artefacts of the rainout shelters on micrometeorology were minor. The heat wave in August 2003 across Europe, which could have interfered with our experiment, occurred several weeks after the shelters were already removed (shelter removal in June 2003). Hence, the heat wave influenced both control and drought plots in the same way. Consequently, the impact of the heat was only important for the comparison between the two years, as soil moisture, soil respiration and annual above-ground productivity were significantly lower in 2003 compared to 2002.

The modeled annual soil respiration losses of slightly above 1 kg C m^-2^ yr^-1^ in our 19 grasslands (for control plots in 2003) fit well to previously reported soil respiration fluxes for a wide range of European grasslands, e.g., mean losses of 1108 g C m^-2^ yr^-1^ given by Bahn et al. (2008). Thus, our approach of data assimilation and modeling seems very robust and appropriate to test our original objectives.

Assessing soil respiration in the 19 different grasslands varying in above-ground productivity and species richness over two years, showed that soil respiration was driven by annual above-ground productivity but not by species richness. Annual above-ground productivity influenced soil respiration in both years, while this effect was strongest within-site. We assume that a high annual above-ground productivity leads to high rates of below-ground plant C allocation which in turn promoted soil respiration, either directly by root respiration or indirectly by increased availability of substrates for microbial respiration. Thus, our results support previous findings where grassland soil respiration had been related to gross primary productivity on an annual scale [9], but also on shorter time-scales [13, 31–33].

The drought treatment significantly reduced soil respiration at all 19 grassland sites. The mean reduction in soil respiration across our 19 sites at the end of the simulated drought period by 20% in 2002 and 34% in 2003 confirms previous studies about drought effects on soil respiration [9, 13, 34–37]. Similarly, annual above-ground productivity was significantly reduced by drought, while species richness and root biomass were unaffected by the drought treatment.

**Fig 5:**
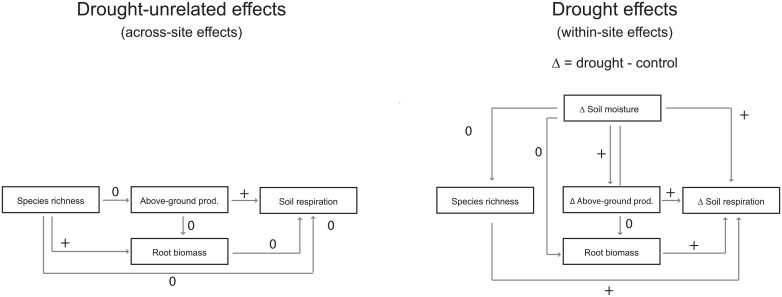
Interactions of biotic drivers with soil respiration and its response to drought. Schematic illustration of the interaction of the investigated biotic drivers (i.e. annual above-ground productivity, species richness and root biomass) with soil respiration irrespective of the drought treatment (left) and with the drought response of soil respiration (right). + indicates positive correlations; 0 indicates no significant effect. Note that ΔSR was always calculated as the difference of soil respiration rates between drought and control plots (SR drought—SR control), thus negative ΔSR values depict reduced soil respiration rates due to the drought treatment and the more negative ΔSR, the larger the drought effect on soil respiration. As an example, species richness is positively correlated with ΔSR under drought conditions, which indicates a more positive ΔSR with increasing species richness, i.e. a lower drought effect on SR with increasing species richness.

Investigating potential interacting effects of biotic drivers on the drought response of soil respiration (Fig 5), we found several aspects that are highly relevant also in the broader context, e.g. when it comes to the future reaction of grassland ecosystems to drought, the role of grassland ecosystems in carbon cycling or model predictions of climate change effects on soil respiration. First, changes in soil respiration in response to drought at a specific site were positively correlated with drought-induced reductions in annual above-ground productivity. The above-ground productivity level of a site, on the other hand, did not have any influence on the magnitude of the relative reduction in soil respiration. This suggests that the drought response of soil respiration is independent of the above-ground productivity level of a site, but that the reduction in soil respiration at a specific site (within-site) is strongly linked via below-ground plant C allocation to the reduction in above-ground productivity at this site. This tight coupling between above- and below-ground processes is in accordance with previous studies [11–15] and urgently calls for the integration of this link into the response functions of soil respiration to drought.

The most remarkable results of our study was that species richness was related to the drought response of soil respiration (Figs 4 and 5). Soil respiration decreased less at species-rich sites compared to species-poor sites (significant in 2002, trend in 2003), suggesting a stabilization effect of species richness in case of drought which is not in line with results by Vogel et al. [38]. This was the case even though we did not find any direct effect of species richness on soil respiration, in line with De Boeck et al. [25], but in contrast to other studies [23, 24]. Hence, the underlying mechanisms for these biotic interactions with the drought response of soil respiration differ among studies. Across our 19 sites, a strong relationship between root biomass and drought-induced reductions in soil respiration was found: Sites with lowest root biomass showed the strongest relative reduction in soil respiration in response to drought (significant in both years). Since species richness and root biomass were positively correlated, an in-depth analysis revealed that the observed richness effect on the drought response of soil respiration was fully explained by the variation in root biomass. Hence, our results clearly show that sites characterized by high root biomass and high species richness are less drought sensitive than species-poor sites with respect to soil respiration. This is of high importance not only for ecosystem functioning of grassland in general, but also for the role of grasslands in the carbon cycle, since soil respiration returns around 80–100 Pg C per year from ecosystems to the atmosphere globally [1, 39, 40] which accounts for roughly 70% of total CO_2_ release by respiration [41]. Moreover, our results fit well in the context of recent finding showing that the relationship between biodiversity and ecosystem functioning is changed under changed environmental conditions [42].

Whether this observation of biotic effects on the drought sensitivity of soil respiration across our 19 sites was due to an offset of the decreased soil respiration by increased root activity under drought conditions remains unclear. The results of Kahmen et al. [19] within the same experiment support this potential mechanism, as a diversity-dependent increase in root productivity (determined by ingrowth cores) in response to drought was observed at the same sites in 2003. In this earlier study, species-rich sites showed the strongest increase in root productivity. These results also suggest potentially different responses of autotrophic and heterotrophic components of soil respiration to drought, even though an in-depth analysis of these processes lies beyond the explanatory power of our data since we did not partition soil respiration. Results from other studies show diverging results in this aspect: While Balogh et al. [43] reported heterotrophic soil respiration in a dry grassland in Hungary to be less affected by drought compared to its autotrophic counterpart, Heinemeyer et al. [44] found heterotrophic soil respiration in a temperate grassland in the UK to respond more strongly to temperature and moisture than autotrophic soil respiration. Similarly, Joos et al. [35] found a much stronger reduction of the CO_2_ release from litter compared to the one from root respiration and soil organic matter decomposition in response to simulated summer drought in a Swiss lowland grassland. Hence, one explanation for the fact that soil respiration in species-rich grasslands was more drought tolerant than in species-poor grasslands in our study, could be that the more drought-sensitive heterotrophic soil respiration formed a smaller relative fraction of total soil respiration at these sites and that total soil respiration consequently reacts less strongly to drought. Our results further support the hypothesis that ecosystems with high species diversity are more resistant to disturbances than those with low species richness [18–21], which has not been addressed for the response of soil respiration to drought so far. This is of high relevance both for the carbon budget and ecosystem functioning of grasslands under future climate change.

## Conclusions

Our results from 19 temperate grassland sites showed that biotic drivers have a critical effect on the drought response of soil respiration. The reduction of above-ground productivity due to drought has triggered changes in soil respiration in response to drought. Yet, the relative drought effect on soil respiration was found to depend most strongly on root biomass and species richness. Hence, our results imply that soil respiration in species-rich sites characterized by high root biomass is least affected by summer drought. These findings indicate the need to consider also biotic variables, i.e. above-ground productivity, species richness and root biomass, as much as the commonly used abiotic variables, e.g. soil temperature and moisture, for the understanding of soil respiration responses to drought.

## Supporting information

S1 Table Soil respiration (SR) models per plot for each site. Both years were fitted together. The SR function (Eq 1) was fitted for each plot at each site by a constrained non-linear regression, E0 was determined over all data and thereafter kept constant (E0 = 289 K). *SR*_*ref*_, *a* and *b* are the fitted model coefficients. RSS shows the minimized residual sum of square, R^2^ is the squared correlation between measured and modeled soil respiration.
(DOCX)

S1 Dataset Manually measured soil respiration (SR), soil temperature (ST) and soil moisture (SM). (CSV)

S2 Dataset Continuously measured (at 6 of the 19 sites) soil temperature (ST) and soil moisture (SM). (CSV)

S3 Dataset Assimilated continuous soil temperature (ST) data set. (CSV)

S4 Dataset Assimilated continuous soil moisture (SM) data set. (CSV)

S5 Dataset Modelled soil respiration (SR) data set for the 19 sites. (CSV)

S6 Dataset Cumulative soil respiration and biotic parameters. (CSV)
