# Supplementary material for: Effects of plant productivity and species richness on the drought response of soil respiration in temperate grasslands
Source: PLoS One. 2018 Dec 21;13(12):e0209031. doi: 10.1371/journal.pone.0209031 (PMC6303019; doi:10.1371/journal.pone.0209031)
Supplement: S1 Table — Both years were fitted together. The SR function (Eq 1) was fitted for each plot at each site by a constrained non-linear regression, E0 was determined over all data and thereafter kept constant (E0 = 289 K). SRref, a and b are the fitted model coefficients. RSS shows the minimized residual sum of square, R2 is the squared correlation between measured and modeled soil respiration. (DOCX) [file pone.0209031.s001.docx]

S1 Table

| Site | Plot | *SR_ref_* | *a* | *b* | RSS | R^2^ |
| --- | --- | --- | --- | --- | --- | --- |
| 1 | control | 5.00 | 40.06 | 0.73 | 14.68 | 0.71 |
|  | drought | 3.88 | 40.48 | 1.25 | 5.82 | 0.81 |
| 2 | control | 5.00 | 33.99 | 1.45 | 42.42 | 0.54 |
|  | drought | 3.03 | 38.30 | 1.91 | 14.17 | 0.56 |
| 3 | control | 4.07 | 2.85 | 17.44 | 57.00 | 0.31 |
|  | drought | 4.06 | 41.03 | 1.49 | 13.54 | 0.67 |
| 4 | control | 5.00 | 31.79 | 1.25 | 29.96 | 0.75 |
|  | drought | 4.01 | 35.19 | 2.02 | 15.22 | 0.75 |
| 5 | control | 4.48 | 28.41 | 1.76 | 20.88 | 0.69 |
|  | drought | 3.45 | 30.43 | 2.19 | 10.08 | 0.76 |
| 6 | control | 3.72 | 2.74 | 28.12 | 20.98 | 0.73 |
|  | drought | 4.64 | 38.72 | 1.77 | 21.77 | 0.74 |
| 7 | control | 4.61 | 0.72 | 9.02 | 20.83 | 0.79 |
|  | drought | 3.76 | 12.80 | 6.96 | 43.03 | 0.51 |
| 8 | control | 4.02 | -17.09 | 8.48 | 31.81 | 0.75 |
|  | drought | 4.07 | 22.96 | 4.10 | 16.66 | 0.83 |
| 9 | control | 3.21 | 1.08 | 11.16 | 25.30 | 0.76 |
|  | drought | 3.01 | 16.16 | 9.56 | 27.70 | 0.67 |
| 10 | control | 4.07 | 42.73 | 0.69 | 20.33 | 0.82 |
|  | drought | 2.84 | 28.45 | 7.37 | 18.88 | 0.78 |
| 11 | control | 3.57 | -54.63 | 20.57 | 10.57 | 0.88 |
|  | drought | 3.31 | 19.44 | 7.99 | 39.69 | 0.61 |
| 12 | control | 3.58 | 37.02 | 2.89 | 21.75 | 0.79 |
|  | drought | 3.26 | 36.08 | 5.61 | 30.58 | 0.63 |
| 13 | control | 4.13 | 31.69 | 3.04 | 23.82 | 0.88 |
|  | drought | 4.60 | 30.08 | 6.18 | 30.41 | 0.87 |
| 14 | control | 3.24 | 31.66 | 3.64 | 15.18 | 0.86 |
|  | drought | 3.07 | 11.36 | 8.32 | 17.78 | 0.76 |
| 15 | control | 3.89 | 1.90 | 12.47 | 18.84 | 0.81 |
|  | drought | 3.72 | 11.20 | 5.76 | 25.32 | 0.78 |
| 16 | control | 5.00 | 38.08 | 0.86 | 20.01 | 0.87 |
|  | drought | 2.76 | 26.82 | 5.58 | 10.44 | 0.83 |
| 17 | control | 3.06 | 1.98 | 14.60 | 7.51 | 0.76 |
|  | drought | 2.93 | 22.26 | 8.39 | 9.72 | 0.82 |
| 18 | control | 3.15 | 1.43 | 11.46 | 15.74 | 0.82 |
|  | drought | 3.02 | 31.90 | 3.70 | 9.29 | 0.83 |
| 19 | control | 2.82 | 1.74 | 15.28 | 9.32 | 0.84 |
|  | drought | 2.93 | 29.90 | 5.89 | 14.12 | 0.83 |
